# Reply to “Missing Skeletal Muscle Metastases of Papillary Thyroid Carcinoma”

**DOI:** 10.3390/diagnostics10070458

**Published:** 2020-07-06

**Authors:** Liviu Hitu, Calin Cainap, Dragos Apostu, Katalin Gabora, Eduard-Alexandru Bonci, Marius Badan, Alexandru Mester, Andra Piciu

**Affiliations:** 1 Iuliu Hatieganu University of Medicine and Pharmacy, 400012 Cluj-Napoca, Romania; Liviu.Hitu@umfcluj.ro (L.H.); gabora.katalin@yahoo.com (K.G.); bonci.eduard@gmail.com (E.-A.B.); badan_marius@yahoo.com (M.B.); 2 Department of Medical Oncology, Iuliu Hatieganu University of Medicine and Pharmacy, 400012 Cluj-Napoca, Romania; calincainap2015@gmail.com (C.C.); piciuandra@gmail.com (A.P.); 3 Department of Orthopedics and Traumatology, Iuliu Hatieganu University of Medicine and Pharmacy, 400012 Cluj-Napoca, Romania; apostudragos@yahoo.com; 4 Department of Oral Health, Iuliu Hatieganu University of Medicine and Pharmacy, 400012 Cluj-Napoca, Romania

Mr. Laszek Herbowski, we would like to thank you for your time and patience in writing your comment on our article. The fact that the published article caught your attention and opened a comments section is very important to us. I think the most correct approach is to try to answer exactly, in the order pointed out by you.

**Point number one**: we are glad that your letter mentions a positive remark, and that you agree with the comment that this is an exceptionally rare clinical presentation; but immediately you present the data from your paper that also includes patients with follicular thyroid carcinoma. I want to mention that our original article refers strictly to cases of papillary thyroid carcinoma with muscle metastases.

**Point number two**: of course, there are cases of muscle metastases from papillary thyroid carcinoma that are not reported in the literature, I do not see the logic of referring to these cases if we do not have access to them. I analyzed the bibliography presented in the letter and I will comment carefully on each case as briefly as possible.

Pucci et al. [1]: this case was included in our article. 

Tamiolakis et al. [2]: as the title says, the case represented a dissemination of papillary thyroid carcinoma four years after the FNAB (Fine-needle Aspiration Biopsy), which is not considered a metastasis with a natural pathophysiological mechanism. 

Panoussopoulos et al. [3]: this case was included in our article. 

Kim et al. [4]: “the tumor subsequently recurred around the original operative bed and subcutaneous tunnels after tumor rupture during surgery and extraction”, which again cannot be considered an original metastasis.

[5,6,7,8]: all case reports were included in our article as well. 

Krajewska et al. [9]: this title was found on the PubMed database only in the form of an abstract. For meticulous control of the truthfulness of the published data, in our article we included exclusively the full available cases from the PubMed database and ResearchGate. 

[10,11]: articles not available in the PubMed database. 

[12,13]: included in our study. 

Li et al. [14]: as previously specified commenting on references [2,4], this case cannot be considered an authentic metastasis due to iatrogenic origin. 

Mohapatra [15]: this case was also included in our article. 

Morita et al. [16]: although the tumor was resected with parts of the of the surrounding lateral pterygoid, masseter, and temporal muscles, in the final histopathological report it was vaguely presented: “The final pathologic report of the infratemporal fossa lesion was metastatic PTC without lymphatic infiltration. Cancerous tissue was not observed in the right lobe of the thyroid or in the muscular process of the mandible.” Thus, it was not clearly specified which structures in the infratemporal fossa were included in the metastatic lesion. 

Califano et al. [17]: the FNAB result reported ambiguously ”dorsal soft tissue lesion showing papillary thyroid cancer metastases” but it did not specify whether the metastasis was in muscle tissue, adipose tissue, or elsewhere. 

Ceriani et al. [18]: this is a consistent case with clear data that should have existed in our review, I totally agree with you on this reference. 

Madan et al. [19]: article not available in the PubMed database. 

Yun et al. [20]: same as point 18. 

[21,22]: these case reports were included in our study. 

Portela et al. [23]: this title was found on the PubMed database only in the form of an abstract. 

Cassidy et al. [24]: article not available in the PubMed database. 

[25,26]: these case reports were included in our study. 

Baloch et al. [27]: the exact FNAB location was not clearly specified, and the result was unclear: “A fine-needle aspiration showed a tumor with follicular growth pattern, and thyroglobulin immunostain was positive. This morphology and immunoprofile was consistent with metastasis from thyroid primary”.

In conclusion, out of the 27 references presented by you, 13 were included in our study, four did not exist at all in the PubMed database, three had inaccurate histopathological or FNAB results, two were present only as an abstract in the PubMed database, and two cases met the inclusion criteria in our article.

**Point number three**: in your paper there were 27 cases scrutinized, not 34, which is the correct number of your counted metastases, exactly as stated by you in point number two. Further, the data presented by you were from your original paper which had different inclusion criteria for patients compared to our study, which were not relevant to it.

I am confident that the direction of your comment was a constructive one and I hope for future cooperation.
